# Clinical impacts of resection margin status and clinicopathologic parameters on pancreatic ductal adenocarcinoma

**DOI:** 10.1186/s12957-020-01900-0

**Published:** 2020-06-22

**Authors:** Tsengelmaa Jamiyan, Takayuki Shiraki, Yoshihiro Kurata, Masanori Ichinose, Keiichi Kubota, Yasuo Imai

**Affiliations:** 1 Department of Diagnostic Pathology, Dokkyo Medical University, Tochigi, Japan; 2 Department of Gastroenterological Surgery, Dokkyo Medical University, Tochigi, Japan; 3 Department of Surgery, Shioya Hospital, International University of Health and Welfare, Tochigi, Japan; 4 Department of Diagnostic Pathology, Ota Memorial Hospital, SUBARU Health Insurance Society, 455-1, Oshima, Gunma 373-8585 Japan

**Keywords:** Pancreatic ductal adenocarcinoma, PanIN, Resection margin, Differentiation grade, Tumor biology, Prognosis

## Abstract

**Background:**

The clinical relevance of pancreatic intraepithelial neoplasia (PanIN) at the resection margin of pancreatic ductal adenocarcinoma remains unknown. We aimed to investigate its clinical impact at the pancreatic transection margin (PTM) and, based on the result, determine the prognostic values of the resection margin status and other clinicopathologic parameters.

**Patients and methods:**

We retrospectively analyzed 122 consecutive patients who underwent pancreatoduodenectomy or distal pancreatectomy between 2006 and 2018. Pathologic slides were reviewed and survival data were retrieved from institutional databases. Associations between two variables were investigated by Fisher’s exact test. Survival curves were generated by the Kaplan-Meier method. Prognostic factors were assessed using Cox regression analysis.

**Results:**

Tumors were resected without leaving macroscopic remnants. The median follow-up period after surgery was 524.5 days. Cancer-related death (*n* = 72) was marginally and significantly associated with local recurrence (*n* = 22) and distant metastasis (*n* = 79), respectively. Local recurrence and distant metastasis occurred independently. After excluding cases with invasive cancer at any other margin, PanIN-2 or PanIN-3 (*n* = 21) at the PTM did not adversely affect prognoses compared with normal mucosa or PanIN-1 (*n* = 57) with statistical significance. R0 resection (*n* = 78), which is invasive cancer-free at all resection margins, showed somewhat better local recurrence-free and overall survivals as compared with R1 resection (*n* = 44), which involves invasive cancer at any resection margin, but the differences did not reach statistical significance. In contrast, differentiation grade and nodal metastasis were significant predictors of distant metastasis, and tumor location and differentiation grade were significant predictors of cancer-related death. Although there was no significant difference in differentiation grade between the head cancer and the body or tail cancer, nodal metastasis was significantly more frequent in the former than in the latter.

**Conclusions:**

PanINs at the PTM did not adversely affect prognosis and R0 resection was not found to be a significant prognostic factor. Differentiation grade might be an indicator of occult metastasis and affect patients’ overall survival through distant metastasis. In addition to successful surgical procedures, tumor biology may be even more important as a predictor of postoperative prognosis.

## Introduction

In the USA, pancreatic cancer is the third leading cause of cancer death, with an estimated 56,770 new cases and 45,750 deaths in 2019 [1]. The 5-year relative survival rate is 34% for the localized stage, 12% for the regional stage, 3% for the distant stage, and 9% for all stages combined [2]. The vast majority (85%) of pancreatic cancer is ductal adenocarcinoma and approximately 70% of all pancreatic cancer occurs in the head, 20% in the body, and 10% in the tail [3, 4]. Surgery combined with adjuvant therapy offers the best chance for long-term survival, but resection is usually possible in only 15–20% of all patients [5]. The 5-year survival rate amounts to only 7–25% even in patients who underwent surgery [6].

Many clinicopathologic parameters have been raised as possible postoperative prognostic factors, among which resection margin involvement is believed to be critical to longer survival. Macroscopic margin involvement can be avoided with careful preoperative planning and operative procedures, but microscopic margin involvement is often observed unexpectedly. The routinely evaluated resection margins consist of the pancreatic transection margin (PTM), circumferential resection margin (CRM), bile duct margin (BDM), and enteric margins. Intraoperative frozen section diagnosis (FSD) is usually performed in pancreatoduodenectomy (PD) and distal pancreatectomy (DP) to avoid cancer involvement at the PTM. However, pathologists often worry about whether to recommend an additional resection when pancreatic intraepithelial neoplasia (PanIN)-2 or PanIN-3/carcinoma in situ was observed. It is empirically accepted that no therapy is needed for PanIN-1 and PanIN-2 [7], but there is little consensus on the impact of PanIN-3. The purpose of this study was to investigate the clinical impact of PanIN observed at the PTM, and, based on the result, to investigate the prognostic values of cancer-free resection margins and other putative prognostic factors.

## Materials and methods

### Patients

We retrospectively analyzed consecutive patients who underwent surgery for pancreatic ductal adenocarcinoma (PDAC) at Dokkyo Medical University Hospital between 2011 and 2018 and at Shioya Hospital, International University of Health and Welfare, between 2006 and 2018. Fourteen cases of total pancreatectomy, seven cases complicated by malignancies in other organs, two cases that died of surgery-related complications, one case with macroscopic remnant tumor, and one case with uncertain margin status were excluded. As a result, data from a total of 122 patients were analyzed. Patients’ clinicopathologic data were obtained via the electric medical chart systems in each hospital. Patient follow-up was performed every month at the outpatient clinic for 5 years after surgery or until they were referred to other institutions for social reasons or deteriorated performance status. A blood test was performed every 2 months and radiographic imaging studies were performed every 3 months for the first 6 months, every 6 months for 18 more months, and yearly for 3 more years. Postoperative recurrence and metastasis were detected mostly by biochemical markers and radiographic modalities. Diagnoses of peritoneal and pleural metastases were performed by cytological investigation. Local recurrence was defined as the appearance of new mass lesions by contrast-enhanced computed tomography (CT), magnetic resonance imaging, or positron emission tomography-CT within the resection field and pancreatojejunal anastomosis site where tumors could be removed without macroscopic remnants. Therefore, diagnosis of the local recurrence was made irrespective of pathologic resection margin status, whether it was R0 (free of invasive cancer at all margins) or R1 (microscopically involved by invasive cancer at any margin). Metastases to other organs and recurrence in non-regional lymph nodes were categorized as distant metastasis. Re-elevated biochemical markers after surgery without a mass lesion recognizable by imaging modalities were judged as distant metastasis to an unknown site. The study protocol was approved by the institutional ethics review boards of both institutions (approvals R-12-20J and 13-B-316).

### Histopathologic analysis

**Fig. 1:**
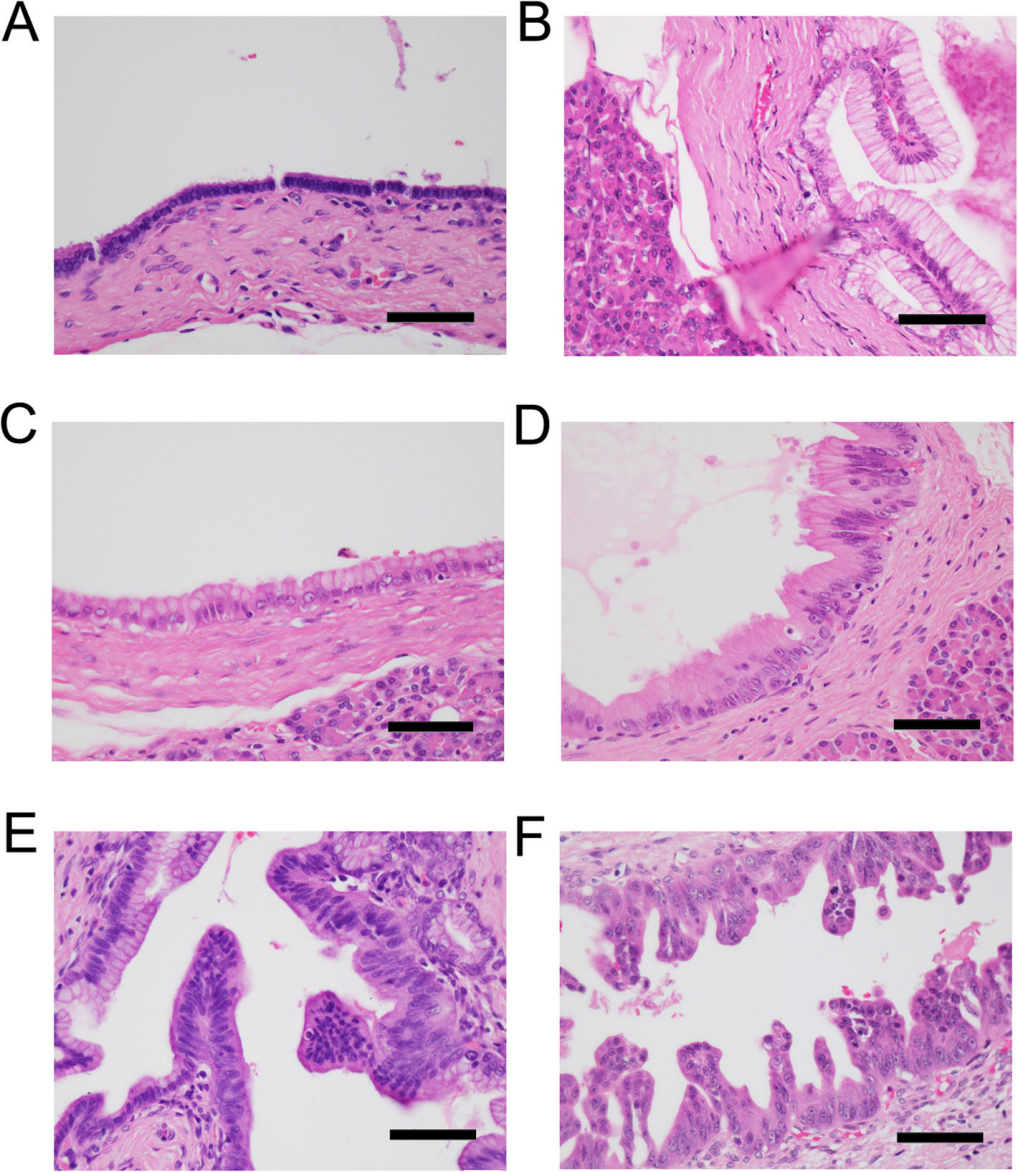
Histopathology of PanIN. **a** Normal pancreatic duct (H&E). **b** Hyperplastic pancreatic duct epithelium (H&E). **c** PanIN-1A (H&E). **d** PanIN-1B (H&E). **e** PanIN-2 (H&E). **f** PanIN-3 (H&E). PanIN, pancreatic intraepithelial neoplasia. Scale bar, 200 μm

Histopathologic diagnosis was performed using the World Health Organization classification of tumours of the digestive system, 4th edition [8]. Representative histopathology of PanIN-1, PanIN-2, and PanIN-3 observed at the PTM are shown in Fig. 1. Because PanIN-2 and PanIN-3 are thought to be truly neoplastic but sometimes PanIN-1 is difficult to discriminate from regenerative atypia [9], combined normal mucosa and PanIN-1 and combined PanIN-2 and PanIN-3 were compared. Stage grouping was performed according to the TNM classification of malignant tumors, 8th edition [9]. All sections were reviewed for confirmation of the original diagnosis by two pathologists (TJ and YI), and they resolved diagnostic discordance through discussion.

Most PD and all DP samples were sliced perpendicular to the main pancreatic duct with modification as advocated by the Japanese Pancreatic Society [6, 10]. In eligible cases, microscopic statuses of PTM and BDM were determined by intraoperative FSD, which was confirmed by comparison with permanent section diagnosis (PSD) that reused frozen samples postoperatively. If discordance between FSD and PSD was observed, PSD was adopted as the final diagnosis. Microscopic statuses of the other PTM and BDM, enteric margin, and CRM were determined by investigating formalin-fixed permanent samples postoperatively. The microscopic statuses of PTM and BDM were determined by examining the surface of the resection margin. The invasive cancer-free and -involved BDMs were designated as BDM0 and BDM1, respectively. The CRM consisted of anterior, medial (uncinate), and posterior margins [6]. CRM with margin clearances of < 1 mm was evaluated as positive for invasive cancer (CRM1) and that with margin clearances of ≥ 1 mm was designated as negative for invasive cancer (CRM0) [11, 12].

To evaluate the clinical impact of the PTM status, survivals of patients with invasive cancer-free resection margins other than PTM were compared according to the PTM status.

### Statistical analysis

Data obtained at the time of surgery were analyzed. Specific parameters between two patient cohorts and associations between two variables were compared using Fisher’s exact test except for age, which was compared using the Mann-Whitney *U* test. Survival curves were generated using the Kaplan-Meier method, and curves were compared by the log-rank test. Postoperative prognosticators were investigated by multivariate analysis. The multivariate Cox regression analysis by forced entry method was performed on parameters with *P* values < 0.10 by the univariate Cox analysis. *P* values < 0.05 were considered significant. Statistical analysis was performed using IBM SPSS Statistics 25 (IBM, Armonk, NY, USA).

## Results

### Prognoses of patients

The patients consisted of 65 (53.3%) males and 57 (46.7%) females aged 43 to 90 years old, with a median age of 69. Out of the patients, 71 (58.2%) and 51 (41.8%) underwent PD and DP, respectively. Pathologic examination of resected specimens revealed that 48 (39.3%), 50 (41.0%), 19 (15.6%), and 5 (4.1%) patients were stage I, II, III, and IV, respectively. Neoadjuvant chemotherapy, consisting of gemcitabine and/or S-1 (tegafur/gimeracil/oteracil), was performed in 65 (53.3%) patients. Adjuvant chemotherapy, consisting of gemcitabine, S-1, FOLFILINOX, or nab-paclitaxel, was performed in 106 (86.9%) patients. Seven (5.7%) patients received irradiation for the treatment of local recurrence or distant metastasis postoperatively. The overall follow-up periods from surgery to cancer-related death or censoring were 68 to 2772 days, with a median of 524.5 days. Tumors were resected without leaving macroscopic remnants in all analyzed cases. Local recurrence was observed in 22 (18.0%) of 122 patients. Distant metastasis was observed in 79 (64.8%) patients: liver in 32 (26.2%), lung in 14 (11.5%), pleura in 5 (4.1%), peritoneum in 25 (20.5%), non-regional lymph node in 14 (11.5%), bone in two (1.6%), and unknown site in two (1.6%) patients. Local recurrence and/or distant metastasis were observed in a total of 90 (73.8%) patients. Cancer-related death was observed in 72 (59.0%) patients. The survival time of patients who died of cancer was 96 to 1435 days, with a median of 455 days. The Kaplan-Meier curves showed that 3-year and 5-year local recurrence/distant metastasis-free survival rates were both 18.7%, and 3-year and 5-year overall survival rates were 35.8% and 23.1%, respectively. Clinicopathologic findings of the patients are summarized in Table 1.

**Table 1:** Clinicopathologic features of the 122 patients with PDAC

Parameters	Patients with PDAC (*n* = 122)
Median age (range)	69 (43–90)
Sex	
Male/female	65/57
Tumor location	
Head/body and tail	71/51
Differentiation grade	
G1/G2/G3/G4	54/52/15/1
TNM stage	
I/II/III/IV	48/50/19/5
Tumor size	
pT1/pT2/pT3/pT4	24/80/18/0
Microvascular invasion	
Positive/negative/unknown	106/14/2
Lymphatic permeation	
Positive/negative	90/32
Perineural invasion	
Positive/negative	102/20
Nodal metastasis	
pN0/pN1/pN2	49/52/21
Neoadjuvant chemotherapy	
Yes/no	65/57
Adjuvant chemotherapy	
Yes/no/unknown	106/12/4
Postoperative radiotherapy	
Yes/no	7/115

*PDAC* pancreatic ductal adenocarcinoma, *G1* well-differentiated carcinoma, *G2* moderately differentiated carcinoma, *G3* poorly differentiated carcinoma, *G4* undifferentiated carcinoma

Distant metastasis was observed in 68 (68.0%) out of 100 patients without local recurrence and 11 (50.0%) out of 22 patients with local recurrence (odds ratio, 0.471 [95% confidence interval (CI), 0.185–1.199]; *P* = 0.140). Local recurrence and distant metastasis occurred independently. Cancer-related death was observed in 55 (55.0%) of 100 patients without local recurrence and 17 (77.3%) out of 22 patients with local recurrence (odds ratio, 2.782 [95%CI, 0.952–8.127]; *P* = 0.060). Finally, cancer-related death was observed in 8 (18.6%) out of 43 patients without distant metastasis and 64 (81.0%) out of 79 patients with distant metastasis (odds ratio, 18.667 [95%CI, 7.206–48.357]; *P* < 0.001). Cancer-related death was marginally associated with local recurrence and was significantly associated with distant metastasis.

### Resection margin status and prognosis

**Fig. 2:**
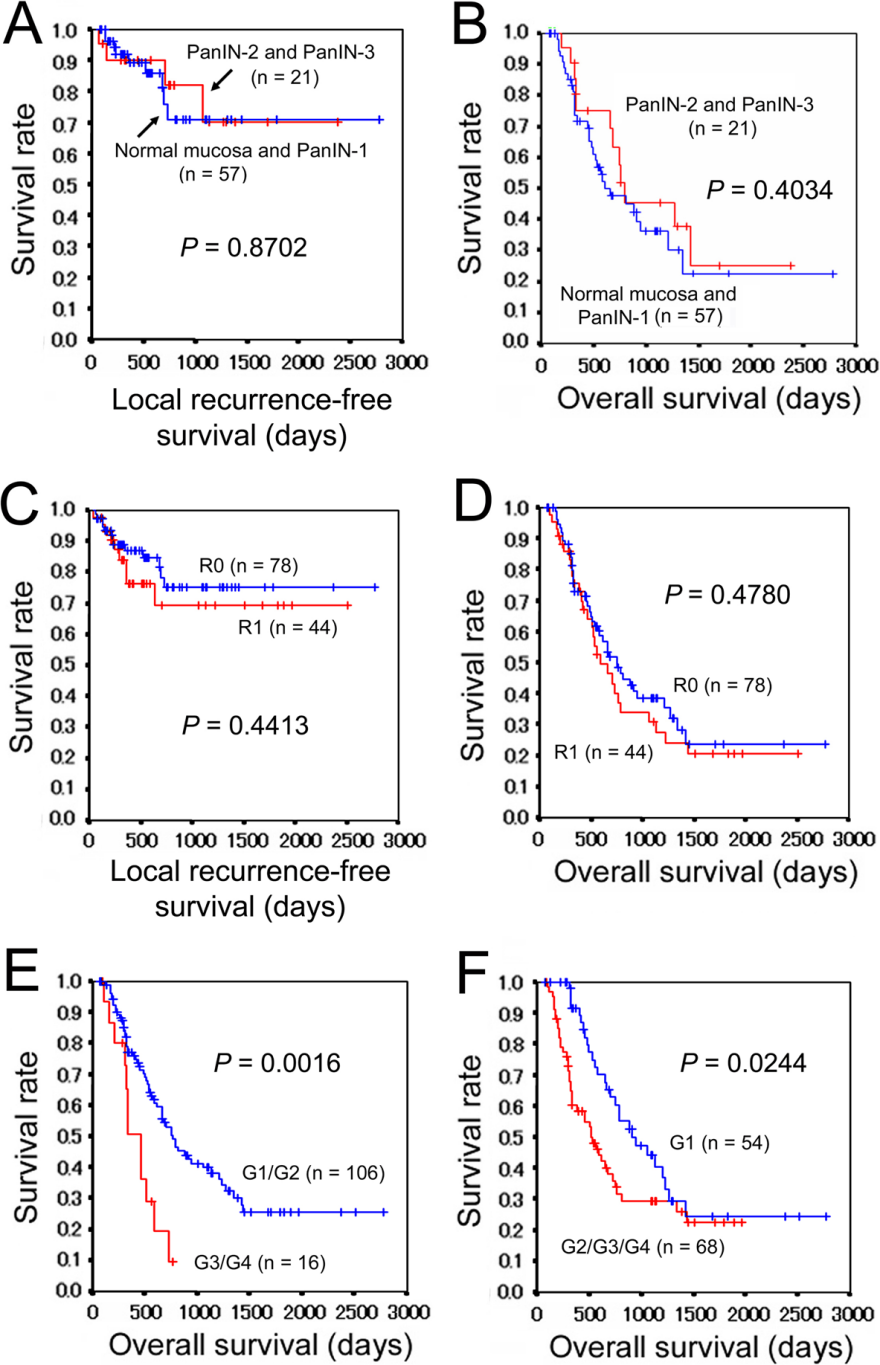
Survival analyses of patients with PDAC. **a** Local recurrence-free survivals of patients with normal mucosa or PanIN-1 and PanIN-2 or PanIN-3 at the PTM. The resection margins other than PTM are invasive cancer-free. **b** Overall survivals of patients with normal mucosa or PanIN-1 and PanIN-2 or PanIN-3 at the PTM. **c** Comparison of local recurrence-free survivals according to the resection margin status. **d** Comparison of overall survivals according to the resection margin status. **e** Comparison of overall survivals according to the differentiation grade of PDAC (G1/G2 versus G3/G4). **f** Comparison of overall survivals according to the differentiation grade of PDAC (G1 versus G2/G3/G4). PDAC, pancreatic ductal adenocarcinoma; PanIN, pancreatic intraepithelial neoplasia; PTM, pancreatic transection margin; G1, well-differentiated carcinoma; G2, moderately differentiated carcinoma; G3, poorly differentiated carcinoma; G4, undifferentiated carcinoma

Intraoperative FSD of BDM and PTM was performed in 45 (36.7%) and 38 (31.1%) cases, respectively, and concordance rates between FSD and PSD were 97.8% and 84.2%, respectively. One (0.8%) patient was diagnosed as BDM1. Eighty-two (67.2%) patients were diagnosed as CRM0, and 40 (32.8%) patients were diagnosed as CRM1. Enteric margins were negative for cancer in all PD cases. At the PTM, 67 (54.9%) patients were diagnosed as negative, 15 (12.3%) patients as PanIN-1, 16 (13.1%) patients as PanIN-2, and 14 (11.5%) patients were diagnosed as PanIN-3; 10 (8.2%) patients were found to have invasive cancer at the PTM. Cases for each PanIN status at the PTM presented with various microscopic CRM and BDM statuses. When cases with BDM1 and CRM1 were excluded, negative (hereafter, normal mucosa), PanIN-1, PanIN-2, and PanIN-3 were noted at the PTM in 48, 9, 11, and 10 cases, respectively. Clinicopathologic features of cases with normal mucosa or PanIN-1 (57 cases) and those with PanIN-2 or PanIN-3 (21 cases) are listed in Table 2. No significant differences were found in the clinicopathologic parameters between the two groups. Then, local recurrence-free and overall survival curves were generated by the Kaplan-Meier method. There were no significant differences in survival curves between both groups (*P* = 0.8702 and 0.4034, respectively) (Fig. 2a, b). These results suggested that the existence of PanIN-2 or PanIN-3 at the PTM might not adversely affect prognosis. Accordingly, PTM0 and PTM1 were designated as not involving and involving invasive cancer, respectively.

**Table 2:** Clinicopathologic features of patients with normal mucosa or PanIN-1 and those with PanIN-2 or PanIN-3 at the PTM

Parameters	Normal mucosa and PanIN-1 (*n* = 57)	PanIN-2 and PanIN-3 (*n* = 21)	*P* value
Median age (range)	69 (45–85)	69 (56–90)	0.257
Sex			
Male	31	7	0.128
Female	26	14	
Tumor location			
Head	33	15	0.308
Body and tail	24	6	
Differentiation grade of PDAC			
G1/G2	50	19	1.000
G3/G4	7	2	
Differentiation grade of PDAC			
G1	26	11	0.619
G2/G3/G4	31	10	
TNM stage			
I/II	49	18	1.000
III/IV	8	3	
Tumor size			
pT1/pT2	50	20	0.437
pT3/pT4	7	1	
Microvascular invasion			
Positive	52	18	0.383
Negative	4	3	
Unknown	1		
Lymphatic permeation			
Positive	39	15	1.000
Negative	18	6	
Perineural invasion			
Positive	47	15	0.346
Negative	10	6	
Nodal metastasis			
pN0	22	9	0.797
pN1/pN2	35	12	
Neoadjuvant chemotherapy			
Yes	36	9	0.127
No	21	12	
Adjuvant chemotherapy			
Yes	50	19	1.000
No	5	1	
Unknown	2	1	
Postoperative radiotherapy			
Yes	2	2	0.292
No	55	19	

Patients with invasive cancer at any resection margins other than the PTM are excluded. *PanIN* pancreatic intraepithelial neoplasia, *PTM* pancreatic transection margin, *PDAC* pancreatic ductal adenocarcinoma, *G1* well-differentiated carcinoma, *G2* moderately differentiated carcinoma, *G3* poorly differentiated carcinoma, *G4* undifferentiated carcinoma

R0 resection was determined as cases with BDM0 (in PD cases), PTM0, CRM0, and negative enteric margins, while R1 resection meant that these conditions were not satisfied. Based on these criteria, 78 (63.9%) patients were diagnosed as R0, and 44 (36.1%) patients were diagnosed as R1. R1 was expected to be strongly associated with local recurrence. However, local recurrence was observed in 13 (16.7%) of 78 R0 cases and 9 (20.5%) of 44 R1 cases, without a statistical significance (odds ratio, 1.286 [95%CI, 0.500–3.305]; *P* = 0.629). In contrast, distant metastasis was observed in 50 (64.1%) of 78 R0 cases and 29 (65.9%) of 44 R1 cases, without a statistical significance (odds ratio, 1.083 [95%CI, 0.498–2.353]; *P* = 1.000). Cancer-related death was observed in 44 (56.4%) of 78 R0 cases and 28 (63.6%) of 44 R1 cases, without a statistical significance (odds ratio, 1.352 [95%CI, 0.632–2.891]; *P* = 0.451). Local recurrence-free and overall survival curves were drawn by the Kaplan-Meier method according to the R status. Although the R1 cases revealed somewhat more unfavorable local recurrence-free and overall survivals as compared with the R0 cases, the differences did not reach a statistical significance (*P* = 0.4413 and 0.4780, respectively) (Fig. 2c, d).

### Prognostic factors

As a way to screen explanatory variables of prognosis, univariate Cox analysis was performed. Differentiation grade was raised as a candidate of prognostic factor for local recurrence. Tumor location, differentiation grade, lymphatic permeation, and nodal metastasis were raised as candidates of prognostic factors for distant metastasis. Age, tumor location, tumor size, differentiation grade, lymphatic permeation, nodal metastasis, and TNM stage were raised as candidates of prognostic factors for cancer-related death. Resection margin and neoadjuvant/adjuvant chemotherapies were not raised as candidates of prognostic factors for local recurrence, distant metastasis, and cancer-related death. In the multivariate analysis, TNM stage and lymphatic permeation were excluded because TNM stage is determined based on tumor size and nodal metastasis, and nodal metastasis is the result of lymphatic permeation. Multivariate analysis of local recurrence-free survival could not be performed because the only candidate prognostic factor in the univariate analysis was differentiation grade. Differentiation grade and nodal metastasis were found to be significant predictors of distant metastasis, and tumor location and differentiation grade were found as significant predictors of overall survival. These results are summarized in Table 3. The overall survival curves according to the differentiation grade under two manners of dichotomization are presented in Fig. 2e, f.

**Table 3:** The Cox regression analyses of predictive factors

	Local recurrence		Distant metastasis		Overall survival	
	HR (95% CI)	*P* value	HR (95% CI)	*P* value	HR (95% CI)	*P* value
Univariate analyses						
Age (≥ 65)	0.891 (0.379–2.095)	0.791	1.382 (0.845–2.260)	0.197	1.598 (0.964–2.651)	0.069
Sex (male)	0.790 (0.342–1.823)	0.581	0.992 (0.630–1.565)	0.974	0.767 (0.483–1.219)	0.262
Tumor location (head)	1.247 (0.532–2.924)	0.612	1.567 (0.980–2.507)	0.061	1.564 (0.972–2.519)	0.066
Tumor size (pT3/pT4)	1.937 (0.646–5.808)	0.238	1.213 (0.601–2.448)	0.591	1.959 (1.037–3.698)	0.038
Differentiation grade (G3/G4)	3.130 (1.133–8.651)	0.028	2.330 (1.186–4.577)	0.014	2.710 (1.423–5.162)	0.002
Differentiation grade (G2/G3/G4)	1.854 (0.773–4.448)	0.167	1.750 (1.098–2.790)	0.019	1.717 (1.066- 2.764)	0.026
Microvenous invasion (yes)	3.136 (0.422–23.334)	0.264	2.014 (0.874–4.640)	0.100	1.471 (0.672–3.223)	0.334
Lymphatic permeation (yes)	1.278 (0.472–3.466)	0.629	2.273 (1.224–4.224)	0.009	1.911 (1.026–3.558)	0.041
Perineural invasion (yes)	4.169 (0.560–31.015)	0.163	1.060 (0.559–2.012)	0.858	1.136 (0.582–2.219)	0.708
Resection margin (R1)	1.396 (0.595–3.273)	0.443	1.048 (0.651–1.687)	0.846	1.188 (0.738–1.912)	0.479
Nodal metastasis (yes)	1.024 (0.437–2.399)	0.956	2.082 (1.262–3.433)	0.004	1.574 (0.967–2.563)	0.068
TNM stage (III/IV)	1.007 (0.340–2.980)	0.990	n.d.	n.d.	1.768 (1.035–3.018)	0.037
Neoadjuvant chemotherapy (yes)	1.196 (0.516–2.772)	0.677	0.865 (0.550–1.361)	0.531	0.981 (0.617–1.560)	0.935
Adjuvant chemotherapy (yes)	0.867 (0.202- 3.728)	0.848	0.872 (0.377–2.015)	0.748	0.622 (0.284–1.364)	0.236
Postoperative radiotherapy (yes)	1.339 (0.313–5.739)	0.694	0.780 (0.314–1.941)	0.593	0.758 (0.305–1.884)	0.551
Multivariate analyses						
Age (≥ 65)	n.d.	n.d.	n.d.	n.d.	1.481 (0.875–2.506)	0.143
Tumor location (head)	n.d.	n.d.	1.253 (0.770–2.039)	0.364	1.621 (0.988–2.661)	0.056
Tumor size (pT3/pT4)	n.d.	n.d.	n.d.	n.d.	1.586 (0.805–3.124)	0.182
Differentiation grade (G3/G4)	n.d.	n.d.	2.705 (1.353–5.408)	0.005	2.372 (1.228–4.582)	0.010
Nodal metastasis (yes)	n.d.	n.d.	2.125 (1.258–3.590)	0.005	1.260 (0.757–2.099)	0.374
Age (≥ 65)	n.d.	n.d.	n.d.	n.d.	1.603 (0.940–2.736)	0.083
Tumor location (head)	n.d.	n.d.	1.320 (0.809–2.155)	0.267	1.710 (1.033–2.832)	0.037
Tumor size (pT3/pT4)	n.d.	n.d.	n.d.	n.d.	1.902 (0.974–3.717)	0.060
Differentiation grade (G2/G3/G4)	n.d.	n.d.	1.687 (1.057–2.692)	0.028	1.780 (1.091–2.905)	0.021
Nodal metastasis (yes)	n.d.	n.d.	1.857 (1.101–3.132)	0.020	1.140 (0.678–1.918)	0.621

*HR* hazard ratio, *CI* confidence interval, *G1* well-differentiated carcinoma, *G2* moderately differentiated carcinoma, *G3* poorly differentiated carcinoma, *G4* undifferentiated carcinoma, *n*.*d*. not determined

Of note, nodal metastasis was observed in 49 (69.0%) of 71 PDAC in the head and 24 (47.1%) of 51 PDAC in the body and tail, with a significant difference between them (odds ratio, 2.506 [95%CI, 1.189–5.279]; *P* = 0.016). Tumor location was not significantly associated with the differentiation grade (data not shown).

## Discussion

PanINs are atypical proliferative lesions of duct columnar epithelium in the smaller pancreatic ducts (< 5 mm in diameter). PanINs were divided into three grades based on the degree of architectural and nuclear atypia, such as PanIN-1, PanIN-2, and PanIN-3. It is postulated that PanINs go through a stepwise progression from PanIN-1, to PanIN-2, to PanIN-3, and finally to PDAC [7]. Recent large-scale genome analyses of PDAC reconfirmed that most recurrent genetic aberrations are *KRAS*, *CDKN2A*, *TP53*, and *SMAD4* [13–15]. Low-grade PanIN (PanIN-1 and PanIN-2) frequently bears activating *KRAS* mutations and inactivating mutations or epigenetic silencing of the *CDKN2A* gene [16, 17]. In addition, high-grade PanIN (PanIN-3) and PDAC further accumulate inactivating mutations of *TP53* and *SMAD4* [16, 17]. These genetic features also suggest that PanIN is a precursor of PDAC.

PanIN is often encountered at the PTM [18]. PanIN-1 is often indistinguishable from non-neoplastic regenerative change. PanIN-2 and PanIN-3, which are thought to be truly neoplastic, often coexist in various proportions, and it is often difficult to differentiate between PanIN-2 and PanIN-3 in the setting of intraoperative FSD. We therefore investigated the prognostic effects of PanIN-2 and PanIN-3 together. As far as we know, there is only one report discussing the clinical relevance of PanINs left behind in the PTM. Matthaei et al. reported that PanIN-3 at the PTM (15 cases) had no impact on overall survival of patients who underwent R0 resection [19]. In this study, we also did not observe an adverse effect of PanIN-2 or PanIN-3 on local recurrence-free and overall survivals. These data suggest that PanIN at the PTM does not affect the postoperative prognosis. It also means that an additional resection might not be needed when PanIN-2 or PanIN-3 is faced at the PTM upon intraoperative FSD. It has been shown that progression from preinvasive precursor lesions to invasive PDAC occurs over many years or decades, and the time required for a parental pancreatic cancer to gain the capacity to invade and metastasize is typically more than 5 years [20]. The 3-year survival rate among our cases was 35.8% and the 5-year survival rate was 23.1% after surgery. Residual PanIN would not have enough time to affect prognosis compared with metastatic PDAC. In line with this speculation, Konstantinidis et al. reported that PanIN was incidentally discovered in 26% of patients who underwent resection for non-adenocarcinomatous lesions but that presence of any grade of PanIN did not result in appreciable cancer risk in the remnant pancreas after adequate follow-up (median 3.7 years, range 0.5–12.6 years) [21]. Another possible explanation is that dispersed, discontinuous growth of PDAC that might have existed across the PTM might have diluted the prognostic effect of PanIN-3 [6, 22]. The greatest limitation of this study is the small sample size. Based on the FSD during surgery, additional resections are usually performed when not only invasive cancer but also PanIN are observed at the PTM. Therefore, we could not collect a large number of cases with PanINs at the PTM. In addition, some cases had invasive cancers in other resection margins, and this further reduced our sample size. Cooperation of multiple high-volume centers would be required to resolve this issue.

We also could not observe a significant prognostic effect of the R status, although the R1 patients revealed somewhat worse local recurrence-free and overall survivals than the R0 patients. Among the resection margins, CRM is generally believed to be most frequently involved [6]. In this study, CRM1 was defined as the presence of an invasive cancer < 1.0 mm from the surface. The resection margin status based on the 1-mm rule has been reported to be a significant prognosticator in local recurrence and/or cancer-related death [23–26]. However, there are also some contradictory studies that have failed to demonstrate a benefit on overall survival for patients with margin-negative resections [27, 28]. We speculate that these contradictory results might be partly explained by a restricted number of included cases and varying procedures for margin analysis in different institutions. In addition, there has been a controversy over the definition of microscopic resection margin involvement. A microscopically negative resection margin was considered as the absence of tumor cells at the surface of the resection margin in the USA until 2016 [29], while the resection margin is now regarded as involved if tumor is present in < 1 mm of the margin in Europe and the USA since 2017 [11, 12]. This definition was inconsistent with the definition of PTM1 and BDM1, because PTM and BDM were sectioned and examined parallel to the resection margins and measuring the exact margin clearance was impossible. Although we defined CRM1 as the presence of invasive cancer < 1.0 mm from the surface, CRM with a margin clearance ≥ 1 mm and CRM with a margin clearance of 0.1 to < 1 mm did not show a significant difference in local recurrence-free survival (unpublished observations). Various margin clearances to define CRM0, such as 1.5 mm or 2.0 mm, have been proposed [24, 28, 30]. If CRM with a margin clearance ≥ 1.5 mm or more was designated as CRM0, it might be a significant prognostic factor. Resection margin status would be expected to directly affect local recurrence. In addition, reported local recurrence rates are between 67 and 86% after pancreatic resection with curative intent [6], but in the present study, local recurrence was noted only in 18.0% of patients and its rate was as low as 26.7% by the Kaplan-Meier method. This smaller number of clinical endpoints may also explain the low power to detect prognostic significance.

Some researchers have observed prognostic significance in poorly differentiated adenocarcinoma and other authors did so in moderately and poorly differentiated adenocarcinoma compared with better differentiated adenocarcinoma [25, 26, 30–34]. We also observed that differentiation grade was a significant predictor for distant metastasis and cancer-related death. The prognostic relevance of differentiation grade was analyzed under two manners of dichotomization, and both demonstrated a statistical significance in distant metastasis-free and overall survivals. PDAC commonly has a combination of well-formed glands, as well as individual cells and clusters, and it presents with a highly dispersed, discontinuous growth pattern [6, 22]. A high proportion of patients with pancreatic cancer die soon even after complete resection [30]. Autopsy studies that assessed the pattern of recurrence reported that more than 80% of patients who have potentially curative resection develop liver metastasis, with no evidence of local recurrence [35, 36]. These studies suggest occult metastasis present at the time of surgery even in patients staged as having loco-regional disease. In this study, distant metastasis was significantly associated with cancer-related death, and differentiation grade was significantly associated with distant metastasis and cancer-related death. We speculate that differentiation grade might be an indicator of occult metastasis and affect patients’ overall survival through distant metastasis.

The prognosis of PDAC has been reported to be worse when the tumor is located in the pancreatic body or tail, compared to being located in the pancreatic head [37]. This was most likely due to the lack of early symptoms due to biliary obstruction when the PDAC is in the body or tail. In the case of resectable PDAC, however, the 5-year survival rate was better with PDAC in the tail than when it was in the head, according to a report from the National Cancer Database, American College of Surgeons Commission on Cancer, and American Cancer Society [38]. In the present study, overall survival of patients with PDAC located in the head was significantly worse than patients with PDAC in the body and/or tail. The pancreatic head is adjacent to many important organs and blood vessels. Differences of surgical approach may have an impact on the patients’ gastrointestinal function which has a distinct complication that can determine patient outcomes. In addition, it has been postulated that tumor biology might be different between PDAC in the head and that in the body or tail [39, 40]. When PDAC is located in the body and tail, even larger tumors can be resected more frequently and successfully than PDAC in the head [41]. Several studies showed that PDAC in the body and tail has less frequent nodal involvement [32, 42]. Proximal tumors show more dedifferentiation in spite of their smaller size [42]. Consistent with the above hypothesis, nodal metastasis was significantly more frequent in PDAC in the head than in PDAC in the body and tail in this study, but tumor location was not associated with the differentiation grade.

Today, it is generally accepted that adjuvant chemotherapy is beneficial to patients with resectable PDAC [4]. However, adjuvant chemotherapy was not found to be a significant prognostic factor by the multivariate Cox analyses in this study. In addition, although patients who received adjuvant chemotherapy (106 cases, 86.9%) revealed a considerably better overall survival as compared with patients who did not (12 cases, 9.8%) by the Kaplan-Meier method, the difference did not reach a statistical significance (unpublished observation). We speculate that this small number of patients who did not receive adjuvant chemotherapy may explain the failure to detect therapeutic merit with a statistical significance. In addition, 57 (53.8%) out of 106 patients who underwent adjuvant chemotherapy had received neoadjuvant chemotherapy. This may have caused the resistance to anticancer drugs used in adjuvant chemotherapy.

In conclusion, cancer-related death was marginally and significantly associated with local recurrence and distant metastasis, respectively, but local recurrence and distant metastasis occurred independently. PanINs at the PTM did not adversely affect prognosis and R0 resection was not found to be a significant prognostic factor. Differentiation grade and nodal metastasis were significant predictors of distant metastasis, and tumor location and differentiation grade were significant predictors of cancer-related death. Differentiation grade might be an indicator of occult metastasis and affect patients’ overall survival through distant metastasis. In addition to successful surgical procedures, tumor biology may be even more important as a predictor of postoperative prognosis.

## Data Availability

The datasets used and analyzed in the current study are available from the corresponding author on reasonable request.

## Abbreviations

PTM: Pancreatic transection margin
CRM: Circumferential resection margin
BDM: Bile duct margin
FSD: Frozen section diagnosis
PD: Pancreatoduodenectomy
DP: Distal pancreatectomy
PanIN: Pancreatic intraepithelial neoplasia
PDAC: Pancreatic ductal adenocarcinoma
CT: Computed tomography
PSD: Permanent section diagnosis
CI: Confidence interval
R0: Invasive cancer-free at all margins
R1: Involved by invasive cancer at any margin
